# Trapped in the darkness of the night: thermal and energetic constraints of daylight flight in bats

**DOI:** 10.1098/rspb.2010.2290

**Published:** 2011-01-05

**Authors:** Christian C. Voigt, Daniel Lewanzik

**Affiliations:** 1 Leibniz Institute for Zoo and Wildlife Research, Alfred-Kowalke-Straße 17, 10315 Berlin, Germany; 2 Department of Animal Behaviour, Freie Universität Berlin, Berlin, Germany

**Keywords:** locomotion, hyperthermia, energetics, optimal foraging, Chiroptera

## Abstract

Bats are one of the most successful mammalian groups, even though their foraging activities are restricted to the hours of twilight and night-time. Some studies suggested that bats became nocturnal because of overheating when flying in daylight. This is because—in contrast to feathered wings of birds—dark and naked wing membranes of bats efficiently absorb short-wave solar radiation. We hypothesized that bats face elevated flight costs during daylight flights, since we expected them to alter wing-beat kinematics to reduce heat load by solar radiation. To test this assumption, we measured metabolic rate and body temperature during short flights in the tropical short-tailed fruit bat *Carollia perspicillata* at night and during the day. Core body temperature of flying bats differed by no more than 2°C between night and daytime flights, whereas mass-specific CO_2_ production rates were higher by 15 per cent during daytime. We conclude that increased flight costs only render diurnal bat flights profitable when the relative energy gain during daytime is high and risk of predation is low. Ancestral bats possibly have evolved dark-skinned wing membranes to reduce nocturnal predation, but a low degree of reflectance of wing membranes made them also prone to overheating and elevated energy costs during daylight flights. In consequence, bats may have become trapped in the darkness of the night once dark-skinned wing membranes had evolved.

## 1. Introduction

Bats are one of the most successful groups of mammals, dominating mammalian assemblages in tropical ecosystems and only being outnumbered by rodents in species numbers worldwide [1–3]. Yet, foraging activities of bats are predominantly restricted to the hours of twilight and night time, leaving the diurnal aerial niche to birds [4]. To date, three not mutually exclusive hypotheses have been formulated to explain the obligate nocturnal niche of bats: competition with birds [5,6], hyperthermia [7–9] and predator avoidance [10–12]. All of these explanations can be subsumed as risk-averse strategies, suggesting that sporadic daylight flight may occur when survival is more at risk for bats foraging exclusively at night than for bats foraging at both day and night.

Of the three hypotheses, the ‘competition with birds’ hypothesis has received least support. During midsummer, for example, foraging activity of Scandinavian bats partly overlapped with that of potential competitors such as swallows (Hirundinae; Aves), but both groups never hunted in the same aerospace or interacted antagonistically [5]. A survey on the British Isles confirmed that interactions between diurnal foraging bats and birds are rare [13] (but see [6]).

The ‘hyperthermia hypothesis’ presumes that bats are prone to overheating as bat wing membranes are not insulated and therefore short-wave (visible) solar radiation may drive core body temperatures to critical and potentially lethal levels [7–9,12]. Birds, on the other hand, do not overheat because feathers are excellent insulators (e.g. [14]). Laboratory experiments with small European insectivorous bats indicated that flying bats have similar core body temperatures when exposed to darkness or artificial light, which is inconsistent with the idea that light imposes a physiological constraint on bat flight [13]. However, core body temperatures have not been measured for bats flying during the day at natural light and temperature levels.

Most studies so far support the ‘predator-avoidance hypothesis’. For example, island populations of frugivorous pteropodids (flying foxes) and insectivorous vespertilionids have been shown to forage partly at daylight, probably because raptorial predators are absent from these remote places [15–18]. Indeed, model calculations and empirical work indicated that diurnal flights are particularly risky for small insectivorous bats when raptorial predators are present [7,8,13]. On the other hand, vespertilionid bats from Scandinavia foraged in daylight around midsummer, albeit during the darkest part of the day, although raptorial birds occurred in the same area [5]. And even though birds of prey and competing swallows and swifts are missing on the Azorean islands, the insectivorous bat *Nyctalus azoreum* forages mostly at night and only partially at daytime [19], suggesting that other or additional factors than risk of predation or competition are involved in the evolution of bat nocturnality. In summary, clear support for any of the three hypotheses is either weak or ambiguous (reviewed in [20]). Here, we investigated whether bats face thermal and energetic constraints when flying in daylight. In particular, we were interested in metabolic rates and core body temperatures (*T*_b_) of bats flying at natural levels of solar radiation and ambient temperature.

Metabolic rates of flying bats are 8–15 times higher than during rest [21–24]. Owing to these extreme levels of physical activity, flying bats have to dissipate large amounts of heat. In addition to producing endogenous heat during flight, bats also take up heat from their environment. For example, *T*_b_ of flying *Phyllostomus hastatus* increased linearly from about 39°C to 42°C when ambient temperature (*T*_a_) increased from 18 to 31°C, suggesting that bats may face hyperthermia even in darkness or inside a cave when *T*_a_ is high [9]. Absorption of short-wave solar radiation probably adds surplus heat to bats when flying in daylight. In fact, bat wing membranes have a low degree of reflectance (i.e. albedo) and absorb up to 90 per cent of short-wave solar radiation [25,26]. A model calculation predicted that maximum exogenous heat uptake from solar radiation may be eight times higher than endogenous heat production in 20 g bats flying in broad daylight [27]. Thus, bats flying in daylight have to find ways to dissipate heat, or they are prone to the health hazards of hyperthermia. Indeed, a recently suggested hypothesis, the so-called ‘heat dissipation limit theory’, postulates that heat dissipation may be a more general physiological constraint influencing limits of energy expenditures in endotherm animals [28,29].

Overall, bats can make use of two major routes for heat dissipation: respiratory evaporation and cutaneous heat loss. Since neither breathing frequencies nor breath volumes have been shown to vary in bats flying at different *T*_a_ in wind tunnels, respiratory evaporative heat loss seems to account for only a small portion of heat loss (an estimated 14% of total heat loss in *P. hastatus* [9]). Thus, the major route of heat loss must come from cutaneous heat exchange [9]. Since bats lack sweat glands on their wing membranes [30], they cannot cool their bodies via evapotranspiration and chiefly depend on the cooling effect of convective airstreams. Some bat species that fly partly in daylight have thermal spots along their body flanks that most probably dissipate body heat when exposed to solar radiation or high *T*_a_ (*Tadarida brasiliensis* [31]). Besides that, bats are only able to dissipate heat from their dark and naked wing membranes via convective cooling. At low temperatures, this proves to be sufficient for daylight flights—for example during midsummer days (*T*_a_ = 20°C [5]) and in laboratory trials (*T*_a_ = 24°C [13]). Then, *T*_b_ of flying bats remains at normal, sublethal levels [13]. However, bats may be constrained in dissipating sufficient heat if exogenous heat from solar radiation is absorbed additionally by wing membranes and when *T*_a_ is equal to or higher than *T*_b_.

As flying bats flap their wing membranes in complex ways [32], heat absorbance and dissipation are not static during flight. Also, wing tension is known to influence the amount of absorbed heat in that a relaxed wing membrane absorbs more heat than a taut wing membrane [26]. Thus, bats could alter their wing-beat kinematics to reduce the amount of absorbed short-wave solar radiation when flying at high *T*_a_ in daylight. If this is done in a suboptimal way for thrust and lift production, or if bats fly faster to escape direct sunlight, we predicted bats to face elevated flight costs at daytime. This could alter key components of optimal foraging, namely the energy costs of commuting and feeding. As a consequence, bats may be forced into the nocturnal niche when foraging yields a better energy gain at night than at day. We tested this newly postulated ‘elevated flight cost’ hypothesis by measuring the metabolic rate using the Na-bicarbonate method [33] and by determining core body temperatures in tropical short-tailed fruit bats, *Carollia perspicillata* (Phyllostomidae; Chiroptera) when flying for short periods at night and at daytime. We predicted that *T*_b_ and metabolic rates are higher for bats flying at daytime than for bats flying at night.

## 2. Material and methods

### (a) Validation experiments in resting *C. perspicillata*

We chose *C. perspicillata* as our focal species because it is a common fruit-eating bat species with a circumtropical distribution in the Neotropics and an exclusively nocturnal foraging activity [34]. Since previous model calculations predicted that 20 g bats such as *C. perspicillata* should rarely be constrained by hyperthermia close to the equator [7,12], we expected no adverse effects on health from potential hyperthermia during daylight.

We used the Na-bicarbonate method to determine the rate of CO_2_ production (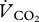) in flying bats as outlined by Voigt *et al*. [35]. We first performed a validation study with 11 male *C. perspicillata*. In March 2010, we captured bats between 17.00 and 22.00 h using 6 and 9 m mist nets (2.5 m height, Ecotone, Gdynia, Poland) in the vicinity of La Selva Biological Station in Costa Rica (10°25′ N, 84°00′ W). After marking bats individually with split forearm bands (A.C. Hughes Ltd, Middlesex, UK, size XB), we transferred them into flight cages (1 m^3^) where they were either kept individually or in groups of two for a maximum of 8 days at ambient conditions and at the natural photoperiod. All bats were released close to their site of capture after the end of experiments. For validation, we compared 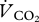 as measured with the Na-bicarbonate method and with 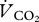 as measured by respirometry. To assess 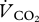 based on Na-bicarbonate, it is essential to estimate the total bicarbonate pool size. For this, we conducted a dilution experiment according to Speakman & Thomson [36] and Hambly *et al*. [37]. Stable carbon isotope ratios were measured using a gas bench coupled to an isotope ratio mass spectrometer (Delta V Advantage, Thermo Finnigan, Bremen, Germany) as outlined in Voigt *et al*. [35]. The ratio of ^13^C : ^12^C was measured in relation to a reference gas, which was calibrated to international standards (NBS 22 and Vienna-Pee Dee Belemnite = *PDB*) and expressed in the *δ* notation (‰). Precision was better than ±0.1‰ (1 s.d.). We converted the *δ* values into atom per cent (AP^13^C; %) according to Slater *et al*. [38] and plotted the natural logarithm of CO_2_ (mol) added to the vacutainers against the natural logarithm of AP^13^C. We then calculated a least-squares linear regression to predict ln(CO_2_) (mol) based on ln(AP^13^CE). AP^13^CE is the enrichment in ^13^C above baseline levels in atom per cent. We calculated the total bicarbonate pool, *N*_c_ (mol) as2.1
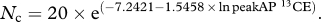


The numbers −7.2421 and −1.5458 refer to the regression equation of the dilution experiment. We included the factor 20 in equation (2.1) to compensate for the 20-fold difference used for the dilution and for the animal experiments.

To assess whether CO_2_ production rates (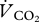) measured by indirect calorimetry matched those estimated by the Na-bicarbonate method, we conducted respirometric measurements with bats exposed to 15°C (*n* = 5) and to 30°C (*n* = 6) in a flow-through set-up. Before each experiment, we weighed the bats to the nearest 1 mg using a precision electronic balance (PM-100, Mettler, Switzerland). A single bat was then placed in a custom-built 3-l respirometry chamber in which temperature was kept constant using a water bath. CO_2_ and water vapour were scrubbed off from the inlet air through tubes filled with ascarite and drierite. Then, air was flushed through the chamber at a rate of 1.8 l min^−1^ as measured by a mass flow controller (Bronkhorst, Melsungen, Germany). A subsampler routed a portion of the outlet air to the cavity ring-down spectrometer (G1101 CO_2_ Isotopic Analyser, Picarro, Sunnyvale, CA, USA). This instrument measured concentrations of ^13^CO_2_ and ^12^CO_2_ (ppmV), in addition to *δ*^13^C (‰) in the outlet air stream of the respirometry chamber.

All animals received 200 mg of 30 per cent (mass/mass) honey water before we injected 200 mg of the aqueous ^13^C-enriched Na-bicarbonate solution intraperitoneally (IP) using a sterile needle and syringe. Immediately afterwards, animals were returned to the respirometry chamber. Then, we monitored changes in ^13^CO_2_ enrichment in relation to ^12^CO_2_ over the following 60 min. We focused on a 15 min period 5 min after peak enrichment, because previous studies indicated a linear washout of the label within this period. For each 1 min period of this interval, we calculated 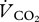 by multiplying the combined concentrations of ^13^CO_2_ and ^12^CO_2_ (ppmV) with the flow-through rate in the chamber using eqn (10.5) from Lighton [39] and assuming that bats exclusively oxidized sugar (respiratory exchange ratio = 1.0). For the same 1 min periods, we calculated the fractional isotopic turnover according to2.2
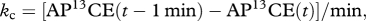
where AP^13^CE was the ^13^C enrichment above background at time *t* and at time *t* − 1 min*. k*_c_ (min^−1^) was multiplied by the total body bicarbonate pool *N*_c_ (mol) and converted to 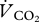 (ml min^−1^).

We used two different methods to calculate the total body bicarbonate pool (*N*_c_) of bats: the plateau and the intercept method [40]. When using the plateau method, we referred to the maximal post-injection enrichment of ^13^C in exhaled breath expressed in AP^13^CE (%) for equation (2.1). When using the intercept method, we extrapolated the isotope elimination curve to *t* = 0 min (i.e. the intercept with the *y*-axis), based on least-squares linear regression equations calculated for the natural logarithm of isotopic enrichments between 5 and 15 min after peak enrichment. We then inserted this intercept into equation (2.1) to calculate the estimated total body bicarbonate pool *N*_c_. For each experimental run, we averaged 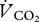 of 1 min intervals measured by indirect calorimetry and 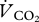 of 1 min intervals measured by Na-bicarbonate. All 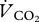 values were converted to standard temperature and pressure conditions.

We plotted 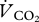 based on respirometry and on the Na-bicarbonate method, and calculated least-squares linear regressions for the two datasets with the plateau and intercept approaches separately. After confirming that the datasets fulfilled the requirements for regression analysis, we tested whether the slopes and intercepts of the regression lines differed from those of the line of equivalence and also whether they differed among each other using Student's *t*-tests.

We estimated the number of bicarbonate body compartments involved during isotopic elimination for resting *C. perspicillata* by calculating the reaction progress variable (1 − *F*) according to Cerling *et al*. [41] and the equation2.3
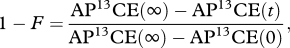
where AP^13^CE (∞) equals the ^13^C enrichment above baseline after infinite time and AP^13^CE(0) the ^13^C enrichment above baseline at time 0. We plotted the natural logarithm of (1 − *F*) against post-injection time and assessed the linearity of the relationship by inspection.

### (b) Flight experiments in *C. perspicillata*

After validation experiments, we released 5 of the 11 bats and captured 4 additional males to minimize the time animals spent in captivity. Flight experiments were similar to the validation experiments except that we allowed bats to fly for 1.5 min approximately 21 min post-injection. Before performing flight experiments with one bat at a time, we allowed each bat to fly in the flight cage so that they would get accustomed to the cage interior. Shortly before the experiments, the animals were fed with 200 mg 30 per cent (m/m) honey water to ensure that bats would oxidize mostly carbohydrates during flight [42]. We injected 200 mg of Na-bicarbonate IP and then put the bat into the respirometry chamber as outlined above. At approximately *t* = 20 min post-injection, we opened the chamber and released the bat in a nearby flight cage (3 m length, 2 m width, 2 m height) within 20–30 s. The flight room was illuminated by dim red light at night so that we were able to observe the bats. Experiments in daylight were performed between 10.00 and 15.00 h using the same flight cage. Since the ceiling of the flight cage consisted of a 1 cm^2^ wire mesh screen, bats were exposed to natural sunlight. The time delay between the last breath collection of the pre-flight period and the onset of flight in the cage was recorded to the nearest 1 s using a stopwatch. We allowed bats to fly continuously for a 1.5 min interval. If bats tried to land, we encouraged them to resume flight by approaching them or by gently touching the wall with a hand at a distance of approximately 0.5 m to the bat. The flight duration was also measured with a digital stopwatch to the nearest second. We recaptured bats with a hand-net and then measured core body temperature (*T*_b_) to the nearest 0.1°C by gently putting the thermocouple of a digital thermometer (GTH 1170, Greisinger electronic, Regenstauf, Germany) at least 10 mm into the bat's rectum. After the temperature measurements reached a plateau, we tried to gently move the thermocouple deeper into the rectum to ensure that measured temperatures were representative of the core body temperature. Approximately 80–100 s after the end of the flight period, bats were put back into the respirometry chamber where isotopic enrichment of exhaled breath and 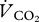 was recorded using the Picarro Isotope Analyser. After a 30 min post-flight period, animals were transferred back to the maintenance cage.

We estimated the isotopic enrichment in the exhaled breath of bats at the onset of flight by calculating a least-squares linear regression on the natural logarithm of AP^13^CE in the exhaled breath of the pre-flight period (3–5 min duration). We defined the pre-flight period as the time point after peak enrichment when the decline of isotopic enrichments turned linear and before we removed the bat from the chamber. By extrapolation, we estimated the isotopic enrichment in exhaled breath when animals started to fly in the flight cage. We used a similar approach for the isotopic enrichment of exhaled breath at the time when bats stopped flying; that is, we used the natural logarithm of isotopic enrichments of the post-flight period (10 min duration) to calculate a regression equation after the least-squares method. The difference in the natural logarithm of AP^13^CE between the pre-flight and post-flight time divided by the flight time yielded the isotope elimination rate of carbon isotopes (*k*_c_) during flight.

Since the validation experiment suggested that 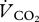 is overestimated when calculated based on *k*_c_ and *N*_c_, we used the factor difference between 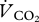 based on respirometry and 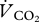 based on isotopic washout to compensate for this overestimation. For this, we calculated 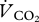 of resting bats during a 10 min post-flight period using both isotopic data and respirometry. We then calculated the ratio between the two 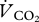 of post-flight resting bats and assumed that this ratio remains constant in flying bats; that is, we multiplied the 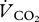 of flying bats based on the Na-bicarbonate method with the ratio derived from post-flight resting bats. To control for the effect of body mass difference between daytime and night-time experiments, we calculated the mass-specific metabolic rate (ml CO_2_ g^−1^ h^−1^).

Environmental variables such as solar radiation and *T*_a_ were recorded during each experiment at a weather station about 200 m away from the flight cage. Solar radiation was averaged over 30 min intervals and accessed via the web page of the station (http://www.ots.ac.cr/meteoro/default.php?pestacion=2 accessed on 15 May 2010).

We calculated Wilcoxon matched-pairs signed-ranks test using GraphPad v. 3 (GraphPad Software, Inc., San Diego, USA) to test for differences in body mass, *T*_b_, and mass-specific metabolic rate when bats flew at night and at day. For all statistics, we used an alpha value of 5 per cent. All values are presented as means ± standard deviation.

## 3. Results

### (a) Validation experiments in resting *C. perspicillata*

**Figure 1.**
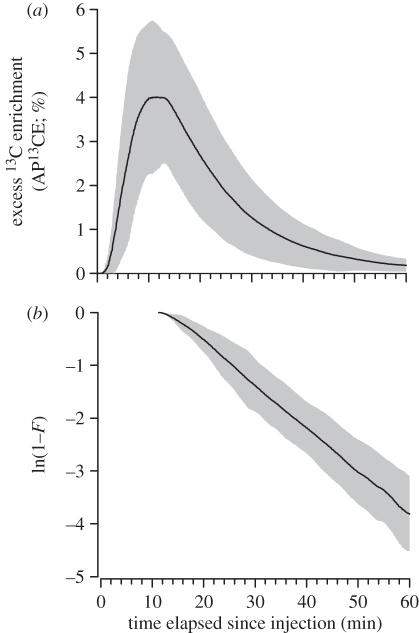
(*a*) Elimination of ^13^C label and (*b*) the reaction progress variable after intraperitoneal injection of 200 mg of ^13^C-labelled Na-bicarbonate in 11 male *C. perspicillata*. The solid line indicates the mean values of ^13^C enrichment above baseline (AP^13^CE; %) and the ln-converted reaction progress variable, respectively. The grey areas indicate the range ±1 s.d.

**Figure 2.**
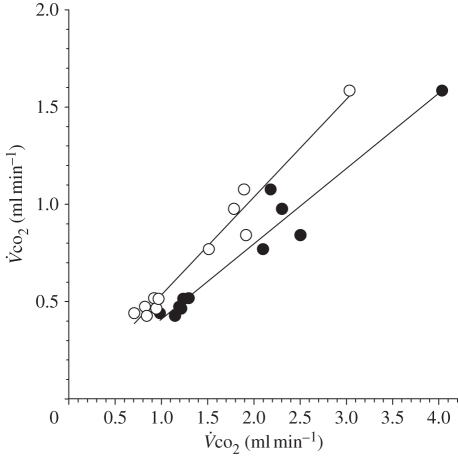
Carbon dioxide production rate (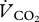; ml min^−1^) measured by indirect calorimetry in relation to 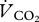 (ml min^−1^) measured by the Na-bicarbonate method. Total bicarbonate pool size was estimated by either the plateau (filled circles) or the intercept method (open circles).

Bats of the validation experiment weighed on average 22.1 ± 1.4 g. Within a few minutes after IP injection of the labelled Na-bicarbonate, the ^13^C label equilibrated in the bats' body bicarbonate pool (figure 1*a*). On average, peak ^13^C enrichments above baseline levels (5.87 ± 1.23 atom%) occurred 11.3 ± 2.4 min post-injection. Intercept values of maximum AP^13^CE equalled 7.37 ± 1.74 atom%. After the peak, *k*_c_ averaged 0.078 ± 0.016 min^−1^ and the dynamics of the reaction progress variable suggested a linear decline, and thus a single pool involved in the washout of the ^13^C label for a 30 min post-peak period (figure 1*b*). The comparison between respirometry and the Na-bicarbonate method revealed that the Na-bicarbonate method yielded higher 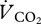 values than the indirect calorimetry (figure 2). 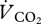 averaged 0.73 ± 0.37 ml min^−1^ when measured with indirect calorimetry, while 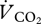 averaged 1.84 ± 0.91 and 1.40 ± 0.71 ml min^−1^ based on the intercept and the plateau approach of the Na-bicarbonate method, respectively. Thus, the Na-bicarbonate method exceeded the respirometric value by 151 ± 26 and 89 ± 18 per cent, respectively. Despite this discrepancy, the coefficient of determination for the relationship between 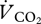 as measured with the Na-bicarbonate method and respirometry was high, equalling 94 and 97 per cent when using the intercept and the plateau method, respectively. Because of the higher *r*^2^ value, we used the plateau method for calculating 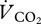 of flying bats.

### (b) Flight experiments in *C. perspicillata*

**Figure 3.**
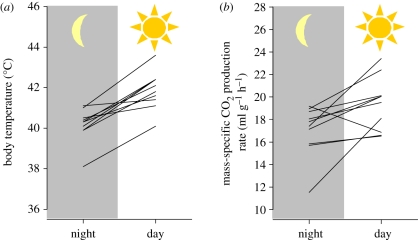
(*a*) Core body temperature (°C) and (*b*) mass-specific CO_2_ production rate (ml g^−1^ h^−1^) in *C. perspicillata* flying at night and during the day.

During experiments in daylight, solar radiation averaged 545 ± 189 W m^−2^ and *T*_a_ 31.5 ± 1.3°C. In contrast, *T*_a_ dropped to 24.5 ± 1.2°C during night-time experiments (*T*_a_ difference: 7.0 ± 2.0°C: 10 pairs; *T+* = 55, *T−* = 0.0; *p* = 0.002). Bats weighed on average 19.5 ± 1.4 and 20.8 ± 1.2 g when flying at night or at daytime, respectively. The difference of 1.3 ± 0.8 g between night and daytime was significant (10 pairs; *T+* = 55, *T−* = 0.0; *p* = 0.002). After flying for 1.5 min at night, *T*_b_ averaged 40.2 ± 0.8°C and CO_2_ production rate 5.53 ± 0.81 ml min^−1^ (*n* = 10 bats). After flying for 1.5 min in daylight, *T*_b_ of post-flight *C. perspicillata* averaged 41.9 ± 0.9°C, a difference of 1.7 ± 0.8°C between night and day (10 pairs; *T+* = 55, *T−* = 0.0; *p* = 0.002; figure 3*a*). Carbon dioxide production rates of diurnally flying bats averaged 6.69 ± 0.72 ml min^−1^ and were higher than when bats flew at night (10 pairs; *T+* = 45, *T−* = 0.0; *p* = 0.004). Since bats flying during the day and at night-time differed in body mass, we calculated mass-specific metabolic rates (ml CO_2_ g^−1^ h^−1^). Mass-specific metabolic rates were significantly higher during daytime flights (19.4 ± 2.4 ml CO_2_ g^−1^ h^−1^) than during night flights (17.0 ± 2.3 ml CO_2_ g^−1^ h^−1^; 10 pairs; *T+* = 49.0, *T−* = −6; *p* = 0.0273), an increase of 15.3 ± 19.0 per cent in mass-specific metabolic rate between day and night (figure 3*b*).

## 4. Discussion

We tested whether bats flying at daytime experienced higher core body temperatures (*T*_b_) and higher metabolic rates than during nocturnal flights. Indeed, we demonstrated that bats encountered a 2°C increase in core body temperature and a 15 per cent increase in mass-specific metabolic rate when flying in daylight than when flying at night. These findings support the hyperthermia and elevated flight-cost hypotheses. To the best of our knowledge, this is the first study to show that *T*_b_ and flight metabolism are elevated when bats fly at natural levels of daylight and *T*_a_.

At night, *T*_b_ of flying *C. perspicillata* was 3°C higher than that of conspecifics resting at about 30°C *T*_a_ [43,44]. Thus, endogenous heat production increased *T*_b_ of flying bats even at night. When flying during daytime, *T*_b_ increased by an additional 2°C to about 42°C. Although this value is in the upper range of physiologically normal *T*_b_, we never observed effects of elevated *T*_b_ in post-flight bats, such as panting or prostration. We conclude from this observation that bats are capable of tolerating excess heat load when exposed for short periods to solar radiation and high *T*_a_. Overall, our data of high *T*_b_ in post-flight *Carollia* are consistent with the hyperthermia hypothesis (i.e. the combined effect of solar radiation and increased *T*_a_ drives *T*_b_ of flying bats higher) [7–9,12]. We argue that bats could nonetheless forage for short periods in daylight (e.g. they could switch between close foraging patches or hunt insects during brief foraging bouts). This could be particularly advantageous when solar radiation is less intense—for example, during cloudy days or under a forest canopy. Why do bats not fly more often in daylight, then?

Metabolic rates were about 15 per cent higher when *C. perspicillata* flew in daylight than when they flew at night. Differences in flight metabolism between diurnal and nocturnal bats have strong implications for optimal feeding strategies of bats. When exposed to sunlight, bats may only be able to dissipate excess heat if *T*_a_ is low, because convective heat loss is a function of the difference between ambient and body temperature. This might explain, for example, why temperate-zone bats are conducting diurnal flights in winter [45] or around midsummer at high latitudes [5]. By contrast, at high *T*_a_ bats may not be able to reduce the combined heat load of muscular work, air temperature and solar short-wave radiation. Then, bats face elevated flight costs, and diurnal flights may only turn profitable if the flight period is short, food reward is high and/or predation risk is low. Higher foraging costs of diurnally active bats may be particularly disadvantageous in situations when bats compete with birds over the same food sources (i.e. a foraging patch may become unprofitable for bats, but may still be profitable to birds, if bats and birds encounter divergent foraging costs).

But some bats from remote ocean islands fly in daylight, for example large pteropodid bats on the Samoan or Comoran islands [16,18]. Since raptorial birds are missing on these islands, the presence of diurnally active pteropodids has long been considered as an argument for the predator-avoidance hypothesis (e.g. [20]). However, flying foxes are more efficient in gliding than small bats [46], and therefore pteropodids may compensate for the higher energy costs of diurnal flapping flight by energy-saving gliding. The insectivorous *N. azoreum*, a vespertilionid bat from the Azorean islands, is also partly foraging at daytime. But, even though hunting *N. azoreum* can be observed in broad daylight, they still forage mostly at night [19], a pattern that is still in line with the hyperthermia and elevated flight-cost hypotheses, but not with the predator-avoidance or bird competition hypotheses.

In general, wing membranes of extant bats have a low reflectance. Yet we do not know whether ancestral bats shared this feature, because wing membranes are not well preserved in the fossil record. It seems likely that dark wings constitute the ancestral state because bats evolved from crepuscular or nocturnal insectivores, which are usually dark-skinned nowadays. Predation probably influenced the behaviour of bats in the Early Eocene. But given that both diurnal and nocturnal predators existed in the Early Eocene, it is not well supported whether predation selected for or against nocturnality when bat flight evolved [11]. Irrespective of the predation scenario in the Eocene, a low albedo made ancestral bats more susceptible to overheating and increased flight costs. Interestingly, global temperatures and incoming solar radiation are thought to have been elevated during the early Eocene when Chiropterans evolved (e.g. [47–49]). This may have aggravated thermal and energetic constraints of ancestral Chiroptera and forced bats into the darkness of night when they conquered the prehistoric skies. It is here important to note that we do not consider the mechanisms mentioned above as exclusive, but rather as complementary or additive driving selective forces for the evolution of bat nocturnality.

Heat absorption would be drastically reduced if ancestral bats had evolved highly reflective or even transparent wing membranes. But white-winged bats may be more prone to predation at night than dark-winged bats at daytime [7,12]. And transparent wing membranes, which are usually thin, may not be optimal for supporting larger body masses given the complex and strong mechanic forces of flapping flight. In extant bats, very few small species have almost transparent wings, such as 4 g *Peropteryx leucoptera* [50].

In conclusion, diurnal foraging activities of modern bats are probably constrained by sunlight-related thermal and energetic parameters, and influenced by predation risk. Resource patches profitable to bats at night possibly turn unprofitable during daytime because of elevated foraging costs. Thus, bats foraging at daytime may not only be more exposed to visually oriented aerial predators but also experience suboptimal foraging conditions. These multiple constraints may have forced bats into the nocturnal niche while this species-rich taxon evolved and spread across ecosystems worldwide.
